# Alaskan Berry Extracts Promote Dermal Wound Repair Through Modulation of Bioenergetics and Integrin Signaling

**DOI:** 10.3389/fphar.2019.01058

**Published:** 2019-09-27

**Authors:** Debora Esposito, John Overall, Mary H. Grace, Slavko Komarnytsky, Mary Ann Lila

**Affiliations:** ^1^Food Bioprocessing and Nutrition Sciences Department, Plants for Human Health Institute, North Carolina State University, Kannapolis, NC, United States; ^2^Department of Animal Science, North Carolina State University, Raleigh, NC, United States; ^3^Department of Food, Bioprocessing, and Nutrition Sciences, North Carolina State University, Raleigh, NC, United States

**Keywords:** skin regeneration, wound healing, Alaskan berries, integrins, proanthocyanidins, epicatechins

## Abstract

Various wild berry species endemic to Alaska and the circumpolar North that exhibit unique medicinal properties have long been appreciated by indigenous Arctic communities. Traditional use of Alaskan berry preparations in the treatment of skin wounds is recorded but has not been scientifically evaluated. Alaskan wild berries feature diverse phytochemical compositions that contain a variety of bioactive polyphenols exhibiting anti-inflammatory, antioxidant, and antimicrobial properties, making them ideal for wound healing interventions and natural anti-aging cosmeceutical formulations. Given increasing interest in identifying biologically active plant constituents for wound care and cosmeceutical applications, the objective of this study was to screen several wild berry species endemic to Alaska and the circumpolar Artic for wound healing and in the crude, polyphenol-enriched, and further fractionated extracts of: *Empetrum nigrum* (crowberry), *Vaccinium uliginosum* (bog blueberry), and *V. vitis-idaea* (low-bush cranberry or lingonberry). A cell migration assay with human dermal fibroblasts (HDFa) was performed to model promotion of wound closure, revealing that bog blueberry extract most actively promoted migration, whereas divergent effects observed with other berry extracts were related to compositional disparities. Lipopolysaccharide (LPS)-stimulated inflammatory response variables measured in RAW 264.7 macrophages [reactive oxygen species (ROS), NO production, prostaglandin-endoperoxide synthase 2 (*COX-2*), and inducible nitric oxide synthase (*iNOS*) expression] were suppressed by most extracts/fractions, but especially bog blueberry and proanthocyanidin (PAC) fractions. Wild berry germplasm contained abundant complex flavonoid structures such as PAC and anthocyanins (ANCs), associated with enhanced repair and inflammatory resolution in these models. Next, underlying mechanisms by which PACs and bioactive metabolites (B2 dimer and epicatechin) could influence wound repair and tissue regeneration were examined. PAC metabolites promoted scratch-wound closure and appeared to exert the highest impacts on early stages of wound healing through stimulating mitochondrial bioenergetics (basal respiration, ATP production, and maximum respiratory capacity) and upregulating expression of important extracellular matrix (ECM) proteins (integrin-ß1 and collagen type I α2 chain). Targeting cellular bioenergetics and integrin-mediated cell–ECM signaling with bioactives from Alaskan wild berries shows considerable therapeutic promise to treat chronic skin wounds and inflammatory skin disorders, as well as more generally to support regenerative healing responses and restore function in a variety of tissue and organ settings after injury or aging.

## Introduction

Wild berries contain an impressive array of bioactive phytochemical compounds, which collectively present a range of biological activities targeting key mechanisms involved in healthy tissue development and aging (Paredes-Lopez et al., 2010; Li et al., 2016). The human health–relevant bioactive properties of wild berries can be primarily attributed to their considerable diversity of polyphenolic constituents, typically exhibiting antioxidant, anti-inflammatory, and antimicrobial capabilities (Seeram, 2008; Nile and Park, 2014). Wild berries contain complex mixtures of polyphenolic compounds in relatively dense concentrations, especially major flavonoid subclasses with well-documented health benefits such as proanthocyanidin (PAC) and anthocyanin (ANC) (Esposito et al., 2014). Interspecific differences, as well as environmental factors such as geographic location and climatic variation, substantially influence the accumulation of berry phytochemicals and thus likely alter their profile of health-related bioactivities (Howard et al., 2003; Lila, 2006; Karppinen et al., 2016).

Our previous work extensively characterized and cross-compared the phenolic profiles of Alaskan wild berry species with commercially cultivated species (Grace et al., 2014), demonstrating key qualitative differences between wild and cultivated berries with regard to phytochemical composition and contents. Wild species demonstrated unique compositional profiles with respect to key polyphenolic classes, especially PAC, and generally exhibited quantitative superiority with regard to total phenolic concentrations. Such compositional diversity is interesting in light of the hypothesis that wild species exhibit robust and distinctive bioactivity profiles due to disparities among major bioactive compounds such as PAC and ANC. These findings prompted further investigation of Alaskan wild berries to identify opportunities for biomedical application and commercial product formulation.

The traditional application of Alaskan wild berry preparations for skin wound care has been documented (Kellogg et al., 2010). Corroborating traditional accounts of their efficacy in skin wound healing, there is widespread literature indication that many other plant-derived compounds that bear similar structural principles likewise exhibit physiological benefits in skin homeostasis, skin inflammation, and related disorders, and in wound healing (Dawid-Pać, 2013; Budovsky et al., 2015; Działo et al., 2016).

Wound healing and skin repair are primary therapeutic targets for regenerative as well as cosmetic interventions, which aim to facilitate restoration of normal tissue architecture and function. Wound repair is a highly complex, multifaceted response, which is temporally and spatially coordinated across multiple stages through cooperative actions of diverse cell types, extracellular matrix (ECM) molecules, cytokines, and growth factors (Walraven and Hinz, 2018). Cell–ECM interactions provide critical feedback during wound healing, which is primarily accomplished through dynamic signaling between ECM proteins, particularly collagens and laminins, with cell surface adhesion receptors such as integrins (Manninen, 2015). Integrins are heterodimeric transmembrane glycoproteins formed by differentially associated 18α and 8β subunits (Schnittert et al., 2018). Human epithelial cells are known to express seven distinct types of integrins: α2β1, α3β1, α5β1, α9β1, α6β4, αvβ1, and αvβ6 (Larjava et al., 2011). In view of their fundamental role in regulating cell–ECM communication and mechanical homeostasis of the ECM, which provides the structural context for cell adhesion, migration, proliferation, and differentiation during epithelial and connective tissue development and repair, integrins represent an exceptionally attractive molecular target for corrective modulation with novel therapeutic agents derived from whole botanical sources or extracts.

The aim of the current study was to explore the influence of compositionally diverse Alaskan berry extracts on critical processes underpinning constructive wound repair and tissue regeneration. We examined the extent to which different berry extract/fraction conditions modulated macrophage inflammatory activation, reflecting the early pro-inflammatory phase of repair, as well as evaluating effects on human skin cell migration into the wound as a reflection of the efficiency of wound closure and granulation tissue formation, employing a monolayer exclusion zone assay to model the rate of wound closure and stimulate migration of human dermal fibroblasts (HDFa) into the wound site. Additional mechanistic investigations were conducted in order to elucidate the molecular pathways through which Alaskan wild berry compounds could effectively modulate gene expression networks regulating inflammatory response, wound repair, and tissue regeneration; to that end, individual bioactive principles were selected for testing in a separate scratch-wound healing assay, and bioenergetic measurements were conducted to determine whether these structures modulated integrin–ECM signaling through cellular metabolic adaptations imposed *via* changes in mitochondrial function.

## Materials and Methods

### Reagents and Materials

Reference compounds procyanidin A2 (PAC-A2), procyanidin B2 (PAC-B2), catechin, and epicatechin were purchased from Chromadex (Irvine, CA, USA). 4-Dimethylaminocinnamaldehyde (DMAC), Folin–Ciocalteu reagent, Sephadex LH-20, and Supelco LC-18 12 ml solid-phase extraction (SPE) cartridges were purchased from Sigma-Aldrich Inc. (St. Louis, MO, USA). All organic solvents were high pressure liquid chromatography (HPLC) grade and obtained from VWR International (Suwanee, GA, USA).

### Berry Sources

Three wild Alaskan berry species, bog blueberry (*Vaccinium uliginosum* L.), crowberry (*Empetrum nigrum* L.), and lingonberry (*Vaccinium vitis-idaea* L.), were handpicked when fully ripe (mid-September 2015) from the greater Fairbanks area, specifically in Skiland (about 10 to 15 miles northeast of Fairbanks, on mostly north-facing slopes with a few scattered tree stands), as well as on Murphy Dome (about 15 miles west of Fairbanks, on mostly northeast-facing slopes without trees) see **Supplementary Material**. Upon collection, berries were immediately frozen and stored at −80°C prior to freeze-drying. Freeze-dried berries were ground immediately prior to extraction.

### Extraction and Polyphenol Enrichment

Ground, freeze-dried berries (8.0 g each) were blended (Waring, Inc., Torrington, CT, USA) with 70 ml acidified 70% aqueous methanol (0.5% acetic acid) for 2 min. The mixture was centrifuged (Sorvall RC-6 plus, Asheville, NC, USA) for 20 min at 4,000 rpm [2,688 relative centrifugal force (RCF)], and the supernatant was transferred to 250 ml volumetric flasks. The pellets were re-extracted two additional times, and the combined extracts from each berry were brought to a final volume of 250 ml. An aliquot of 20 ml from each extract was evaporated and freeze-dried to afford the crude extract. The remaining volume from each berry extract (220 ml) was evaporated to about 25 ml before loading onto two Supelclean™ LC-18 SPE cartridges 12 ml (2 g) preconditioned with methanol and acidified water [0.05% trifluoroacetic acid (TFA)]. The adsorbed extract on the cartridges was washed with 50 ml acidified water to remove free sugars and organic acids before eluting the polyphenols with 100% methanol. The methanol eluate was evaporated and freeze-dried to afford the polyphenol-enriched extracts (370, 220, and 321 mg for bog blueberry, crowberry, and lingonberry, respectively). About 50 mg from each extract was stored for phytochemical analysis and biochemical assays.

### Fractionation of Polyphenol-Enriched Extract

The remaining polyphenol-enriched extracts were dissolved in about 10 ml of 20% methanol in water (0.05% TFA) and applied to a column packed with Sephadex LH-20 preconditioned with the same solvent mixture. Isocratic elution of ANC-enriched fractions, fractions 1 and 2 (Fr 1 and Fr 2), continued until the color of eluate faded, at which time the columns were then washed with 70% acetone in water to collect the third PAC-enriched fraction (Fr 3). Solvents were evaporated, and the remaining aqueous extracts were freeze-dried. Bog blueberry extract afforded Fr 1 (120 mg), Fr 2 (50 mg), and Fr 3 (128 mg); crowberry Fr 1 (80 mg), Fr 2 (39 mg), and Fr 3 (60 mg); and lingonberry Fr 1 (80 mg), Fr 2 (24 mg), and Fr 3 (163 mg). Between approximately 6 and 8 mg of each crude extract and fraction in triplicate were dissolved in 60% methanol in water at a concentration of 5 mg/ml and filtered using a 0.20 µm polytetrafluoroethylene (PTFE) syringe filter (Fisher Scientific, Pittsburg, PA, USA) before further analyses.

### Total Phenolic and PAC Determination

Total phenolics were determined with Folin–Ciocalteu reagent (Singleton et al., 1999). Concentrations were expressed as mg/g extract as gallic acid equivalents based on a gallic acid standard curve. Total PAC was determined colorimetrically using the DMAC method (Prior et al., 2010). A series of dilutions of standard procyanidin B2 dimer were prepared in 80% ethanol ranging from 1 to 100 µg/ml. Blank, standard, and diluted samples were analyzed in triplicate. The plate reader protocol was set to read the absorbance (640 nm) of each well in the plate every minute for 30 min (SpectraMax M3, Sunnyvale, CA, USA). PAC concentration was expressed as mg/g extract procyanidin B2 equivalent.

### ANC Determination *via* HPLC and LC–MS

HPLC analysis for ANC identification and quantification was conducted using an Agilent 1200 HPLC (Agilent Technologies, Santa Clara, CA, USA), according to our previously published protocol (Grace et al., 2014). Quantification was performed using the peak areas recorded at 520 nm to construct the calibration curve for cyanidin-3*-O*-glucoside. liquid chromatography-mass spectrometry (LC-MS) analysis was performed using a Shimadzu liquid chromatography-mass spectrometry ion trap time of flight (LC-MS-IT-TOF) instrument (Shimadzu, Tokyo, Japan) equipped with a Prominence HPLC system. The LC separation was done using a Shim-pack XR-ODS column (50 mm × 3.0 mm × 2.2 µm) at 40°C with a binary solvent system comprised of 0.1% formic acid in water (A) and methanol (B). Compounds were eluted into the ion source at a flow rate of 0.35 ml/min with a step gradient of B of 5–8% (0–5 min), 8–14% (10 min), 14% (15 min), 20% (25 min), 25% (85 min), 5% (35 min), and 5% (40 min). Ionization was performed using an electrospray ionization (ESI) source in the negative ion mode. Compounds were characterized and identified by their MS, MS/MS spectra, and LC retention times and by comparison with available reference samples and our previous analyses (Grace et al., 2014).

### PAC Composition by Normal-Phase HPLC−FLD Analysis

Chromatographic separation of oligomeric PACs was conducted using an Agilent 1200 HPLC with fluorescence detector (FLD) according to the previously reported method (Grace et al., 2014). Separation was performed using a Develosil Diol column, 250 mm × 4.6 mm × 5 μm (Phenomenex). The mobile phase consisted of (A) 2% acetic acid in acetonitrile and (B) 95% methanol, 3% water, and 2% acetic acid using a linear gradient from 0% to 40% B, in 35 min; 40% to 100% B, in 40 min; 100% to 100% isocratic B, in 45 min; and 100% to 0% B, in 50 min. Fluorescence of the procyanidins was monitored at excitation and emission wavelengths of 230 and 321 nm. PACs were identified by comparison with available standards, our previous analyses (Grace et al., 2014), reported literature (Hollands et al., 2017), and LC–ESI–MS. Percentages of PACs were calculated based on peak area measurements, relative to total area measurements.

### *In Vitro* Cell Culture

The mouse macrophage cell line RAW 264.7 (ATCC TIB-71, obtained from American Type Culture Collection, Livingstone, MT, USA) was maintained in Dulbecco’s modified Eagle’s medium (DMEM, Life Technologies, Grand Island, NY, USA), supplemented with 100 IU/ml penicillin, 100 μg/ml streptomycin (Thermo Fisher Scientific, Waltham, MA, USA), and 10% fetal bovine serum (FBS) (Life Technologies, Grand Island, NY, USA), not exceeding 80% confluency at 37°C in a humidified incubator with 5% CO_2_. Primary human dermal fibroblasts isolated from adult skin (HDFa, Invitrogen C-013-5C) were cultured in Medium 106 (Invitrogen M-106-500) with Low Serum Growth Supplement (LSGS, Invitrogen S-003-10) and antibiotic penicillin/streptomycin solution 100 IU/100 μg/ml (Fisher MT-30-002-CI).

### Cell Viability and Dose Range Determination

The cytotoxic activity against HDFa was evaluated by MTT ([3-(4,5-dimethylthiazol-2-yl)-2,5-diphenyl-tetrazolium bromide] colorimetric assay performed according to manufacturer protocol and quantified spectrophotometrically at 550 nm using a microplate reader SynergyH1 (BioTek, Winooski, VT, USA). Inhibition was calculated as a percentage against vehicle control, and absolute IC_50_ values were calculated by non-linear regression using twofold serial dilutions of the treatment samples. Treatment concentrations showing no changes in cell viability compared with that of vehicle control were considered optimal and selected for use in subsequent experimental assays.

### ROS and NO Inhibition Assays

To evaluate treatment effects on *in vitro* reactive oxygen species (ROS) production after lipopolysaccharide (LPS) stimulation, an adapted fluorescent dye protocol was used (Choi et al., 2007). RAW 264.7 macrophages seeded at a concentration of 5 × 10^5^ cells per well into a 24-well plate were incubated overnight at 37°C. Fresh fluorescent medium [50 μM solution of dichlorodihydrofluorescein diacetate acetylester (H_2_DCFDA) in sterile phosphate-buffered saline (PBS), Molecular Probes, Eugene, OR, USA] was added to cells for 30 min. After aspiration of the medium, cells received new medium and 1 μl of treatments (all achieving a final concentration of 50 μg/ml) in triplicate and were stimulated with 10 μl of LPS (from *Escherichia coli* 026:B6) prior to incubation for 24 h, to a final volume of 500 μl per well. Fluorescence of 2′,7′-dichlorofluorescein (DCF) was measured at 485/515 nm (excitation/emission) *via* microplate reader (Synergy H1, Biotech, Winooski, VT, USA). LPS induction (no treatment) and vehicle controls were included for reference, along with a positive control containing dexamethasone (DEX), a well-known antioxidant. ROS concentrations were calculated relative to LPS induction control.

Next, the ability of treatment samples to inhibit LPS-stimulated NO radical formation in RAW 264.7 cells was evaluated *via* colorimetric assay in accordance with a protocol that has been previously reported (Van de Velde et al., 2016). Briefly, 100 μl of cell culture medium was added to 100 μl of Griess reagent (1% sulfanilamide and 0.1% naphthylethylenediamine in 5% phosphoric acid), and the mixture was incubated in the dark at room temperature for 10 min. The absorbance at 540 nm was read (Synergy H1, Biotech, Winooski, VT, USA), and a calibration curve built with serial dilutions of sodium nitrite (R^2^ = 0.990) was used to express results as NO concentration relative to LPS induction control.

### Inflammatory Gene Expression Assay and Real-Time Quantitative PCR Analysis

Effects of enriched extract and fraction treatments on LPS-stimulated inflammatory gene expression (*COX-2, iNOS*) were evaluated in RAW 264.7 cells. Treatments were administered 1 h prior to LPS stimulation (1 μg/ml) for 4 h, followed by RNA extraction and purification, cDNA synthesis, and quantitative polymerase chain reaction (qPCR) analysis. Positive (DEX at 10 μM) and negative (vehicle) controls were included in every experiment, and triplicates were made for all treatments and control conditions. Cells were harvested using TRIzol reagent for extraction of cellular RNA, and spectrophotometric quantification was performed using the SynergyH1/Take 3 Reader (Biotek). cDNA synthesis was conducted with 2 μg of RNA from each treatment sample, using a high-capacity cDNA reverse transcription kit (Life Technologies, CA, USA) according to manufacturer protocol, on an ABI GeneAMP 9700 (Life Technologies).

The resulting cDNA was amplified by real-time qPCR using PowerUp SYBR PCR Master Mix (Life Technologies) according to a protocol previously reported (Esposito et al., 2014). Samples were subjected to a melting curve analysis to confirm the amplification specificity. Changes in target gene expression relative to the endogenous genetic control were determined using the 2^-ΔΔ CT^ method (Livak and Schmittgen, 2001).

### Cell Migration Assay and Fluorescence Imaging Analysis

HDFa was seeded into 96-well Oris plates (Platypus Technologies, LLC, Madison, WI, USA) at a concentration of 3×10^4^ cells/ml and cultured to near confluence in cell monolayers. Cells were labeled with fluorescent dyes (NucBlue Live Cell Stain and CellTracker Red CMTPX, at 1 µM). Once confluence was reached, wound induction through removal of well inserts and growth media exposed a central, cell-free zone initiating cell migration. This was immediately followed by the rapid removal of free cellular debris through single wash with sterile PBS, and addition of fresh growth medium containing various test and control treatments; treatments were administered to cells in groups of four wells per treatment condition and included enriched extracts and fractions (each at a final concentration of 50 μg/ml), blank (cell-free) and vehicle (0.5% ethanol) controls, and a positive control (10% FBS) serving as a reference for robust induction of migration. Cell migration into the central zone was quantitatively assessed by cell-specific fluorescence measurement 0, 24, and 48 h post-wounding at excitation/emission wavelengths of 360/460 and 577/605 nm. Fluorescence image analysis was conducted with the EVOS FL Auto Cell Imaging System (Life Technologies), by capturing bright field and fluorescent images at well centers (for consistency) 0, 24, and 48 h after insert removal. EVOS software analysis allowed for parallel assessment of cell migration during wound closure across treatments in real time, providing an illustration of differential capacities for dermal repair promotion. Wound closure as cell migration rate was calculated relative to vehicle control treatment.

### Scratch-Wound Healing Assay and qPCR Array Transcription Profiling

We additionally employed a scratch-wound closure model to test effects of selected Alaskan berry compounds in the interest of generating a diverse bioactivity profile relevant to wound repair. HDFa seeded in 24-well plates were grown to near confluence in cell monolayers, and identical linear wounds were generated in each well using sterile 100 μl plastic pipette tips. After sterile PBS wash removed free cellular debris, fresh growth medium was added to wells in triplicate per treatment, containing either vehicle control (0.1% ethanol) or treatment compounds [procyanidin B2, epicatechin, 3,4-dihydroxyphenylacetic acid, and 3-(3,4-dihydroxyphenyl)propionic acid, all at 10 μM]. Treated cells were then incubated for 24 h prior to pharmacogenomic profiling of genes associated with wound healing and regeneration. Using RT² Profiler PCR Array PAHS-121Z (Qiagen, Hilden, Germany), we examined expression profiles of 84 genes pertinent to ECM, cell adhesion, inflammatory cytokine/chemokine, growth factor, *MAPK*, *TGFB*, and *WNT* signaling pathways. RNA isolation, template cDNA synthesis and qPCR analysis were performed, and relative gene expression levels were normalized to those of five housekeeping genes [ß-actin (*ACTB*), ß-2-microglobulin (*B2M*), hypoxanthine phosphoribosyltransferase 1 (*HPRT1*), lactate dehydrogenase A (*LDHA*), and large ribosomal protein P1 (*RPLP1*)]. The RT² Profiler PCR Array Data Analysis Tool (v3.5, default settings) was used to generate clustergrams to identify co-regulated genes across treatments, and volcano plots were generated to determine significant expression changes across individual gene targets.

### Measurement of Cellular Bioenergetics

HDFa was seeded into a 24-well XF assay plate (2×10^4^ cells per well) overnight and subjected to real-time measurements of oxygen consumption rate (OCR) and extracellular acidification rate (ECAR), using Agilent Seahorse XF24 Extracellular Flux Analyzer (Seahorse Biosciences, North Billerica, MA, USA). Cells were then transferred to 500 μl of XF assay medium (DMEM without NaHCO_3_, 10 mM glucose, 2 mM pyruvate, pH 7.4) and equilibrated in a non-CO_2_ incubator at 37°C for 1 h. OCR and ECAR were automatically recorded by Seahorse XF24 software v1.8. Basal OCR and ECAR rates were determined by averaging the last four basal measurements. Next, following a triplicate treatment with vehicle (0.1% EtOH) or test compounds as indicated (10 μM), eight subsequent measurements were performed every 15 min. For mitochondrial stress tests, the mitochondrial complex inhibitors were then injected sequentially in the following order: oligomycin (1 μM), FCCP (0.75 μM), and antimycin A/rotenone (1 μM each). Four readings were taken after administration of each inhibitor.

**Statistical analysis.** Statistics were performed using the software GraphPad Prism v7 (GraphPad Software Inc., La Jolla, CA, USA). All data were analyzed by one-way or two-way ANOVA as appropriate. Post hoc analyses were conducted using the Dunnett’s multiple comparison tests at 5% level of significance. All results are expressed as means ± standard error of the mean (SEM).

## Results

### Extraction and Polyphenolic Enrichment of Alaskan Wild Berries

Crude extracts were prepared from freeze-dried whole berries using successive enrichment (Grace et al., 2009; Yousef et al., 2013). Crude extracts were purified using C18 SPE to afford the polyphenol-enriched extracts. These polyphenol-enriched extracts of bog blueberry, crowberry, and lingonberry showed substantial enrichment of total phenolics (20-, 10- and 11-fold), ANC (26-, 7.5-, and 14-fold), and PAC (11-, 6.6-, and 8-fold), respectively. Each berry polyphenol-enriched extract was loaded on a Sephadex LH-20 column and eluted with 20% acidified methanol, affording two fractions (Fr 1 and Fr 2) specifically enriched in ANC. Elution with 70% acetone afforded a third fraction (Fr 3) specifically enriched in PAC. For bog blueberry, ANC constituted 52% (Fr 1) and 70% (Fr 2); for crowberry, ANC constituted 98% (Fr 1) and 77% (Fr 2); and for lingonberry, ANC constituted 14% (Fr 1) and 64% (Fr 2). In Fr 3, PAC represented 22% (bog blueberry), 34% (crowberry), and 52% (lingonberry). The results of total phenolic, ANC, and PAC concentrations for the extracts and fractions obtained from each berry are listed in **Table 1**.

**Table 1:** Polyphenol composition of crude, polyphenol-enriched, and Sephadex LH-20 fractionated extracts from wild Alaskan berries.

	*Polyphenol class*	Crude extract	Polyphenol-enriched extract	Fraction 1 (ANC-enriched)	Fraction 2 (ANC-enriched)	Fraction 3 (PAC-enriched)
Crowberry	*TP^1^*	101 ± 4.7	723 ± 5.8	544 ± 22	598 ± 7.6	692 ± 1.4
	*ANC^2^*	104 ± 6.0	780 ± 19	984 ± 31	774.2 ± 16	41.1 ± 2.2
	*PAC^3^*	30.3 ± 1.9	189 ± 19	19.7 ± 0.5	25.1 ± 1.2	339 ± 11
Bog blueberry	*TP^1^*	52.6 ± 3.0	601 ± 18	403 ± 20	584 ± 22	733 ± 9.0
	*ANC^2^*	18.1 ± 0.8	484 ± 11	522 ± 21	701 ± 20	43.5 ± 4.5
	*PAC^3^*	10.0 ± 0.4	113 ± 6.6	28.3 ± 7.2	90.6 ± 6.6	220 ± 21
Lingonberry	*TP^1^*	70.0 ± 0.4	610 ± 10	160 ± 5.2	518 ± 11	700 ± 9.1
	*ANC^2^*	6.9 ± 0.2	97.0 ± 4.0	140 ± 9.0	696 ± 23	2.4 ± 0.5
	*PAC^3^*	46.0 ± 1.7	364 ± 11	11.2 ± 0.5	20.8 ± 0.4	521 ± 12

^1^TP, total phenolics, measured by Folin–Ciocalteu assay as mg/g dry extract and expressed as gallic acid equivalent.

^2^ANC, anthocyanins, measured by HPLC as mg/g dry extract and expressed as cyanidin-3-glucoside equivalent.

^3^PAC, proanthocyanidins, measured by dimethylaminocinnamaldehyde (DMAC) assay as mg/g dry extract and expressed as procyanidin B2 equivalent.

### ANC Determination and Quantification

**Figure 1:**
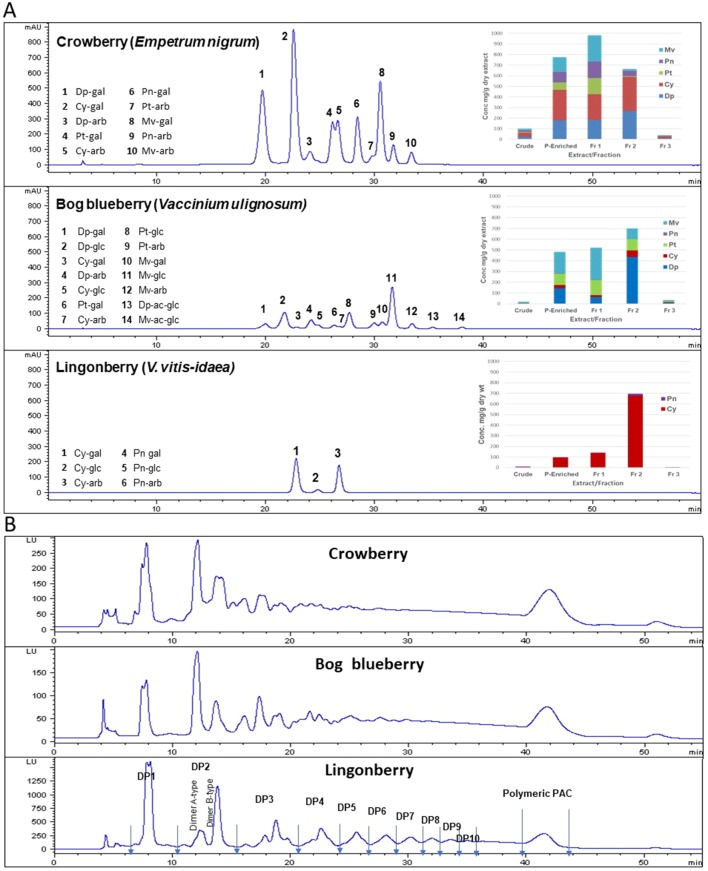
Anthocyanin (ANC) and proanthocyanidin (PAC) compositions of Alaskan berry crude extracts, polyphenol-enriched extracts, ANC-enriched fractions (Fr 1 and Fr 2), and PAC-enriched fraction (Fr 3). Dp, delphindin glycosides; Cy, cyanidin glycosides; Pt, petunidin glycosides; Pn, peonidin glycosides; Mv, malvidin glycosides. **(A)** The HPLC chromatograms presented here represent the polyphenol-enriched extracts, recorded at 520 nm. Concentrations (mg/g dry extract/fractions) were expressed as cyanidin-3-glucoside equivalents. **(B)** HPLC–fluorescence detection (FLD) profiles for proanthocyanidins in fraction 3 from Alaskan berries (excitation, 230 nm; emission, 320 nm).

ANCs were identified based on LC–MS analysis as well as previous reports (Grace et al., 2014) and in comparison with available standards. Quantitative analysis was performed based on HPLC peak areas recorded at 520 nm and expressed as cyanidin-3-glucoside equivalents. Glucosides of malvidin and delphindin represented over 70% of total ANCs present in crude and polyphenol-enriched extracts of bog blueberry. Malvidins were the major ANC in Fr 1 (47% of total ANCs), while delphinidins dominated Fr 2 (62%). Fr 3 contained negligible concentrations of ANC. Crude and polyphenol-enriched extracts of crowberry exhibited high concentrations of cyanidins (37% of total ANC), and delphinidins (24%) as well as malvidins (18%) to lesser extents. Malvidins and cyanidins constituted 50% of total ANC in Fr 1, whereas in Fr 2, 85% of ANCs were delphinidins and cyanidins. Lingonberry exhibited a very distinct ANC profile, in which cyanidins were overwhelmingly predominant throughout all extracts/fractions (>95% of total ANC). **Figure 1** shows the HPLC profile, identified peaks, and concentrations of ANC in each berry extract/fraction.

### PACs Compositional Analysis

**Figure 2:**
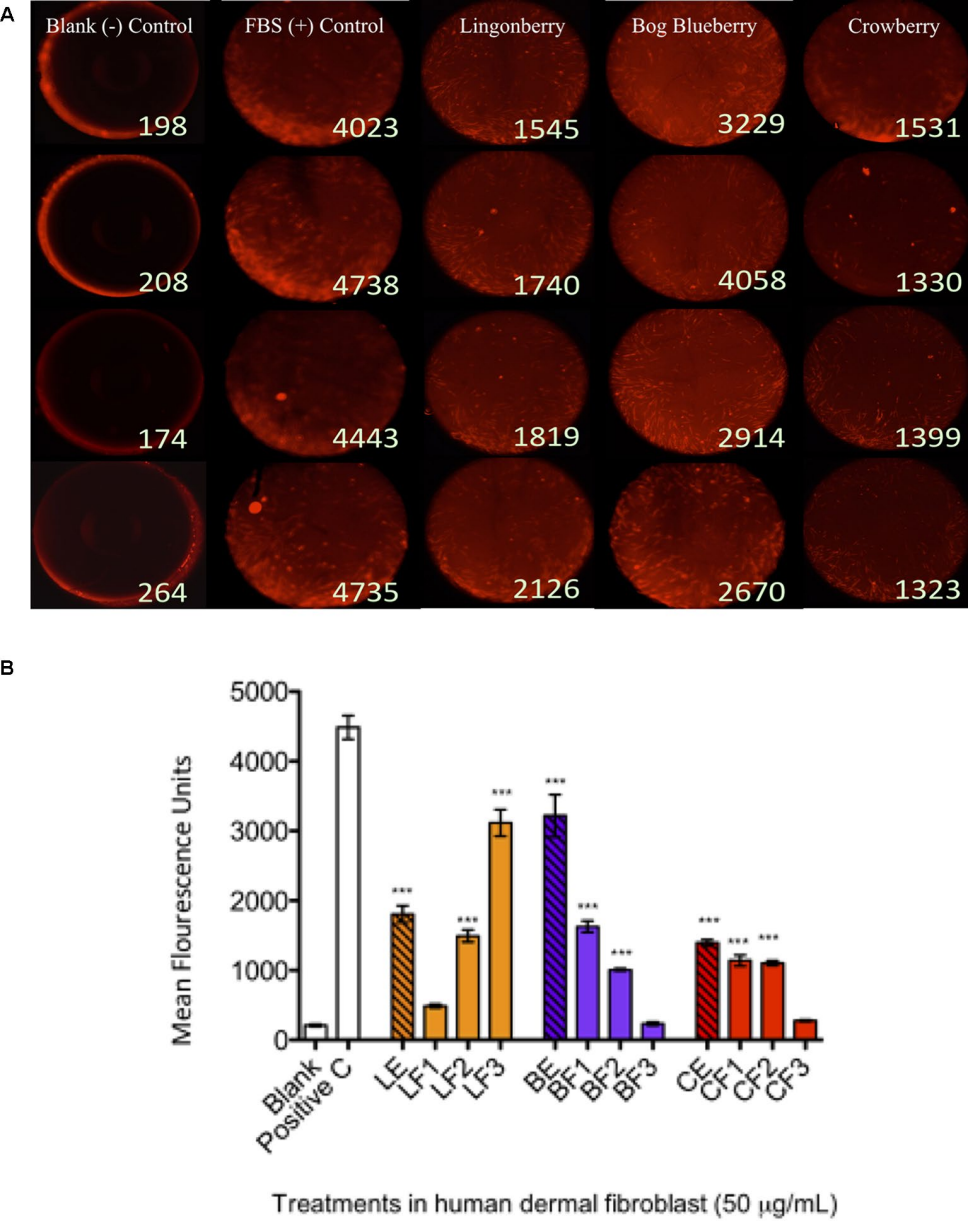
Wound healing assay and cell migration end point detection. Wells were seeded with 50,000 human dermal fibroblast (HDFa) cells. Cells adhered after 18 h and stoppers were removed. All wells received CellTracker Red dye to fluorescently stain the cells prior to 24 h incubation with berry extract treatments to permit cell migration; the fluorescence signals in the detection zones were measured using a plate reader. Pictures demonstrate visualization of cell migration after 24 h treatments in HDFa. **(A)** First column shows the cell-free control (blank); second column shows positive control group [10% fetal bovine serum (FBS)]; third column shows migration associated with bog blueberry (*Vaccinium uliginosum* L.) polyphenol-enriched extract; fourth column shows migration associated with crowberry (*Empetrum nigrum*) polyphenol-enriched extract; fifth column shows migration associated with the lingonberry (*Vaccinium vitis-idaea* L.) polyphenol-enriched extract. All extract treatments were tested at a final concentration of 50 µg/ml. The data represent the average of two experiments ± standard error of the mean (SEM). *P < 0.05; **P < 0.01; ***P < 0.001 (n = 3) using one-way ANOVA and Dunnett’s post-test. All images are taken at a magnification of 4×. Cells are stained with CellTracker CMPTX fluorescence dye. **(B)** The graph illustrates representative cell fluorescence measurements for post-migration (t = 48 h) per treatment (n = 4 wells). *Blank*: cell-free negative control; *Positive C*: FBS positive control; *LE/BE/CE*: lingonberry, bog blueberry, and crowberry polyphenol-enriched extracts, respectively; *F1–F3*: ANC-enriched and PAC-enriched fractions. All extracts were tested at a final concentration of 50 µg/ml.

Normal-phase HPLC with FLD was able to separate proanthocyanin components in the PAC-rich fraction (Fr 3) from the three Alaskan berries according to their degree of polymerization (**Figure 2**). Total peak areas indicated that lingonberry had the highest value, followed by crowberry, and the least was bog blueberry (data not shown); this agrees well with the PAC content measured by the DMAC assay (**Table 1**). All berries contained both type-A and type-B dimers. The normal-phase method cannot not distinguish between the B-types of procyanidins, however our previous study indicated that procyanidin B2 was a common compound in all berries investigated, including Alaskan lingonberry and bog blueberry, utilizing LC-MS on a reversed phase column (Grace et al., 2014). PAC-dimer B constituted 6.09% and 9.30% of total PAC area for bog blueberry and crowberry, respectively, while it constituted 12.3% in lingonberry (**Table 2**). PAC-A dimers constituted 10.41%, 10.18%, and 4.8% for bog blueberry, crowberry, and lingonberry, respectively. These ratios are not consistent with our previous report, which might be due to a different harvest year, where water fall and climate conditions might have been different.

**Table 2:** Analysis of proanthocyanidins oligomers and determination of degrees of polymerization (DPs) in fraction 3 of Alaskan berries by normal-phase HPLC–fluorescence analysis (excitation, 230 nm, emission, 320 nm).

Degree of polymerization	Bog blueberry %*	Crowberry %*	Lingonberry %*
DP1	6.93	10.09	17.95
DP2: Dimer A-type Dimer B-type Total DP2	(10.41) (6.09) 13.07	(10.18) (9.30) 22.78	(4.80) (12.43) 16.57
DP3	13.70	9.53	10.44
DP4	8.59	10.28	8.30
DP5	11.35	6.97	6.81
DP6	5.94	7.08	5.78
DP7	5.64	2.90	4.88
DP8	3.51	4.18	4.20
DP9	3.73	3.76	3.74
DP10	2.86	3.67	3.27
DP11	3.95	2.30	2.93
DP≥12	7.95	4.25	5.67
Polymeric PACs	11.04	12.20	9.46

*Results were calculated based on peak area measurements relative to total area measurements.

### Impacts on LPS-Stimulated Inflammatory Response Variables

Capacities of Alaskan berry treatments (polyphenol-enriched extracts and specifically ANC- or PAC-enriched fractions) to inhibit production of intracellular ROS and NO were investigated in LPS-stimulated RAW 264.7 macrophages. As shown in **Figure 2A**, polyphenol-enriched extract treatments did not reduce ROS production in activated macrophages, whereas ANC- and PAC-enriched fractions (Fr 1–3) were associated with a 25% reduction (p ≤ 0.05), with the exception of F1 of lingonberry. Berry extracts and fractions at 50 µg/ml slightly suppressed NO synthesis (> 25%), as shown in **Figure 2B**, a level of inhibition that was maintained across all treatments. Notably, the highest relative suppression of NO synthesis was achieved by treatment with PAC-enriched fractions of each berry.

The effects of berry extract/fraction treatments on *COX-2* expression are shown in **Figure 2C**. Treatments with bog blueberry polyphenol-enriched extract and all of its fractions induced pronounced inhibition of *COX-2* mRNA expression, particularly in comparison with other berry treatments, which showed weaker impacts. Of exception, the PAC-enriched fractions of lingonberry and crowberry were associated with significant reduction of *COX-2* expression. Interestingly, treatment with the PAC-enriched fraction of bog blueberry produced the greatest *COX-2* inhibition (45%). The impacts of berry treatments on *iNOS* mRNA expression are shown in **Figure 2D**. All berry extracts and fractions significantly attenuated *iNOS* expression (>25%).

### Cell Viability Assay in HDFa and Dose Determination

MTT assay was used to evaluate the viability of HDFa after 24 h incubation with treatments at different concentrations. Alaskan berry extracts in the range of 50–250 µg/ml had nonsignificant effects on cell viability (p > 0.05; data not shown). Based on these data, all subsequent experiments in this work were performed with treatment concentrations of 50 µg/ml.

### Berry Extract/Fraction Effects on HDFa Migration in Exclusion Zone Wound Healing Assay

**Figure 3:**
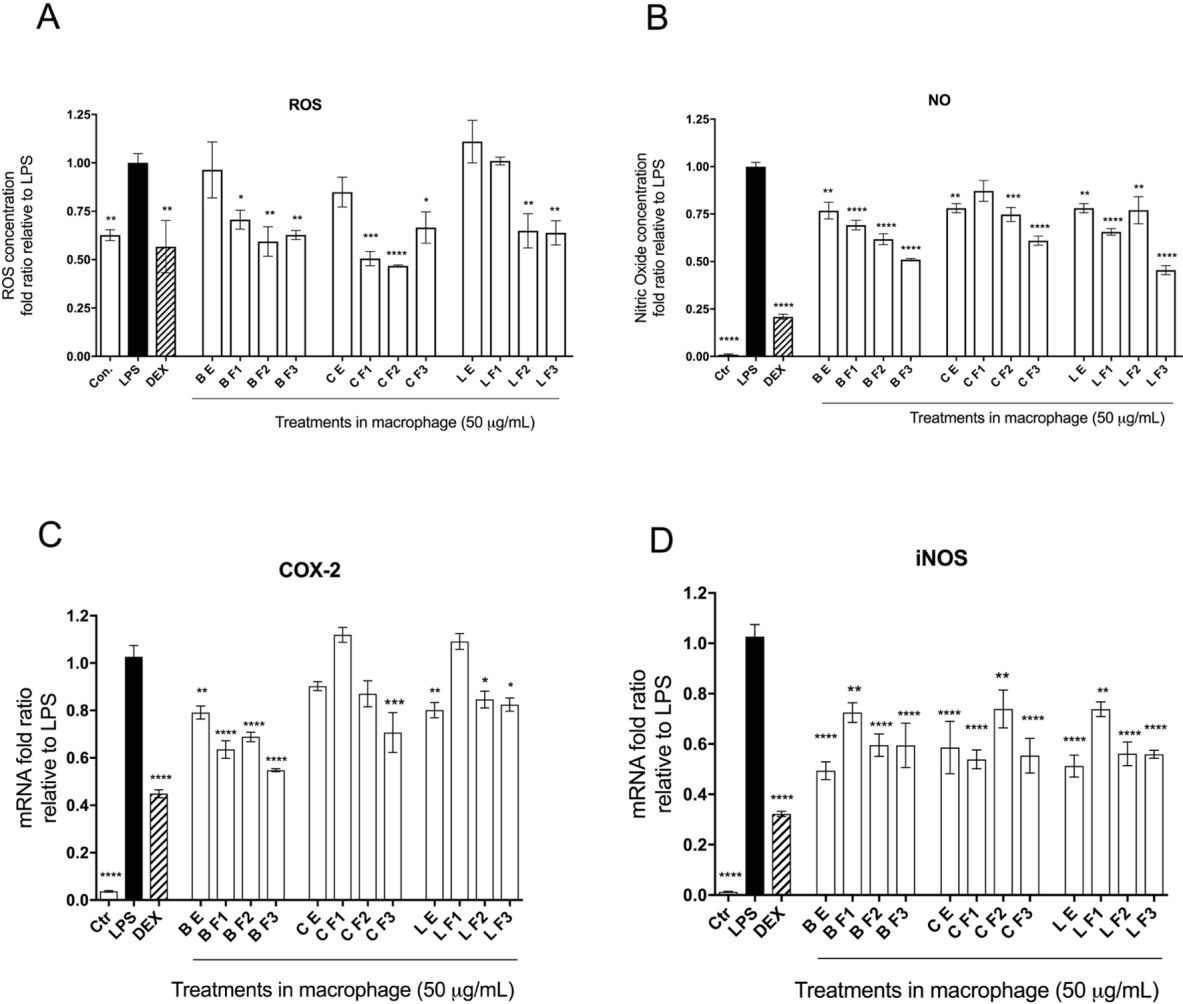
Anti-inflammatory bioactivity of polyphenol-enriched extracts (PE) of Alaskan berries, ANC-enriched (Fr 1, Fr 2), and PAC-enriched (Fr 3). Effects on the expression of the inflammatory biomarker genes in the lipopolysaccharide (LPS)-stimulated RAW264.7 macrophages at 50 µg/ml. Genes involved in the inflammatory response are represented: interleukin *IL-1*ß assay **(A)**, prostaglandin-endoperoxide synthase 2 *COX-2* assay **(B)**, inducible nitric oxide synthase *iNOS* assay **(C)**, and interleukin IL-6 assay **(D)**. Changes in gene expression were measured by comparing mRNA quantity relative to LPS. A value of less than 1.0 indicates transcriptional downregulation (inhibition of gene expression) compared with LPS, which shows maximum genetic induction (1.0). Ctrl: cell treatment with vehicle only; Dex: dexamethasone at 10 µM used as positive control. The data represent the average of two experiments ± standard error of the mean (SEM). *P < 0.05; **P < 0.01 ; ***P < 0.001 (n = 3) using one-way ANOVA and Dunnett’s post-test.

To determine the effects of wild berry extracts on fibroblast migration in skin wound healing, an exclusion zone–based wound healing model (Oris Migration Assay) was conducted with HDFa in a 96-well plate. Prior to cell seeding, each well was populated with inserts (silicone-based stoppers), which inhibit cell adherence to a central zone. After cells seeded and completely adhered around this zone, wounding was induced through removal of inserts to reveal a 2-mm-diameter unseeded area into which migration could proceed. Cell migration could be visualized by fluorescence imaging as cell movement into the unseeded area (**Figure 3A** shows migration at 24 h post-wounding) and rates of migration associated with treatments were quantitatively assessed by measuring increases in cell fluorescence within the central exclusion zone across 48 h (**Figure 3B**), normalized to vehicle control treatment.

In a separate study, phenolic compounds extracted from black soybeans similarly demonstrated the ability to stimulate skin cell migration, an effect that could be attributed to ANC (Nizamutdinova et al., 2009). The authors found that treatment over 48 h with 50 and 100 µg/ml of ANC from black soybean, comprising glucosides of cyanidin (72%), delphinidin (20%), and petunidin (6%), induced human dermal fibroblast and keratinocyte migration *in vitro*; furthermore, black soybean ANC treatment increased the production of vascular endothelial growth factor (VEGF), a key cytokine that stimulates early wound angiogenesis in the initiation of wound repair (Galiano et al., 2004). Thus, ANCs are suggested to potentiate wound healing through facilitating cell proliferation and migration and through increasing proangiogenic VEGF production to accelerate the early stages of wound healing. Another study investigated the effects of several well-known bioactive compounds, including resveratrol, phloretin, and ferulic acid, on the proliferation and migration of human oral fibroblasts (San Miguel et al., 2011). Intriguingly, only high or low (10^−3^/10^−5^M) concentrations exhibited beneficial effects on functional mechanisms regulating fibroblast migration and proliferation during gingival healing or periodontal tissue repair. Evidence from these studies and ours supports the feasibility of bioactive compound delivery to sites of tissue damage/injury as a potential therapeutic strategy to support active tissue repair and encourage regenerative healing through promoting skin cell proliferation and migration. It is virtually certain that the promotion of HDFa migration by enriched and fractionated berry extracts, as observed in the current work, is underpinned by enhanced accumulation of TP, ANC, and/or PAC (**Table 1**), although specific connections between individual classes or compounds with cell migration promotion are unclear.

### Pharmacogenomic Evaluation of Procyanidin B2 and Its Structural Units

**Figure 4:**
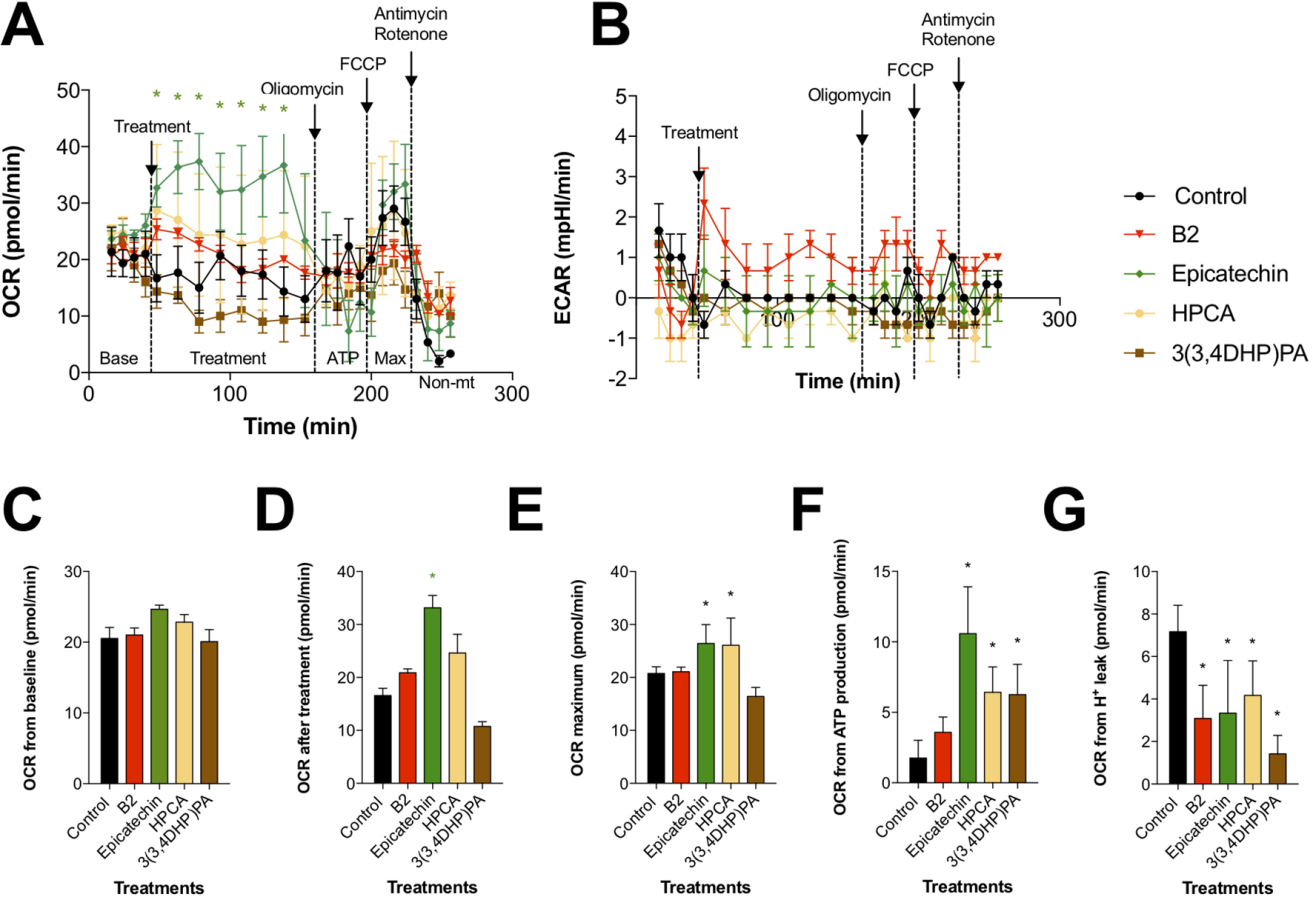
Effects of procyanidin B2 and its metabolites on mitochondrial respiration in HDFa. Changes in **(A)** oxidative phosphorylation and **(B)** glycolysis rates in response to 10 µM of all tested compounds. **(C)** Cells were subjected to six baseline bioenergetics readings, followed by **(D)** eight readings of the corresponding treatments. Next, mitochondrial complex inhibitors were injected to all treatments sequentially, and four readings were taken after each inhibitor. Calculations of **(E)** maximal oxygen consumption rate (OCR), **(F)** OCR from ATP production, and **(G)** OCR from proton leak were expressed as means ± SEM. *P < 0.05 (n = 3).

The phytochemical analysis for PAC composition (**Table 2** and **Figure 1B**) indicated that procyanidin B2 is a common component in the investigated berries. To further elucidate the involvement of procyanidin B2 and its structural metabolites (**Figure 4A**) in wound healing pathways in HDFa, pooled RNA samples were used to measure gene expression changes in ECM and cell adhesion, inflammatory cytokines and chemokines, growth factors, *MAPK*, *TGFB*, and *WNT* signaling pathways in response to each compound. The RT² Profiler clustergram (**Figure 4B**) showed moderate effects of procyanidin B2 on gene expression levels in the HDFa cells, potentiated by epicatechin or homoprotocatechuic acid (HPCA). All structures modulated nearly identical sets of genes, suggesting that they share a structural moiety responsible for mediating the conserved functional effects. 3-(3,4-dihydroxyphenyl)propionic acid clustered together with procyanidin B2, as both compounds showed relatively weaker impacts on repair and regeneration pathways. A volcano plot was constructed from the RT Profiler data (**Figure 4C**). Three genes were upregulated by the majority of treatments: *COL1A2* (pro-alpha2 chain of type I collagen), *ITGB1* (integrin receptor subunit beta 1), and *RHOA* (ras homolog family member A), suggesting that procyanidin-based treatments modulate a complex interplay between ECM proteins (*COL1A2* and *ITGB1*) and *RHO* guanosine triphosphate phosphatase (GTPase) that regulates cell–ECM signaling to support cell adhesion and migration during active tissue repair *via* mechanosensitive reorganization of the actin cytoskeleton (**Figure 4D**).

Another group of prominently downregulated genes across treatments included *MMP2* (matrix metallopeptidase 2) and *CCL2* (C-C motif chemokine ligand 2, also known as *MCP1* [monocyte chemoattractant protein 1]). *MMP2* expression offers a reliable indicator for clinical wound healing and is induced in the inflammatory phase before being suppressed in downstream stages of tissue repair (Karim et al., 2006). Moreover, MMPs are known to mediate aberrant ECM remodeling and potentially contribute to the progressive fragmentation of dermal collagen that is considered as a major driver of skin aging (Qin et al., 2017). *CCL2* promotes monocyte infiltration and macrophage response in wounds early after injury and is similarly suppressed in the subsequent stages of tissue repair (Wood et al., 2014). Reduced expression of both genes strongly suggests that procyanidin and its metabolites may have promoted scratch-wound closure through an acceleration or shortening of the early inflammatory phase. Thus, PAC-based structures from Alaskan berries may impact the trajectory of wound healing and tissue regeneration by promoting earlier inflammatory resolution. Weak simultaneous suppression of multiple genes responsible for the early inflammatory phase of wound healing (*IFNG* [interferon gamma], *IL1B* [interleukin 1 beta], *IL6* [interleukin 6], *HGF* [hepatocyte growth factor], *MIF* [macrophage migration inhibitory factor]) further supports this conclusion.

Among the phenolic metabolites tested, only procyanidin B2 showed moderate suppression of *MAPK3* (mitogen activated protein kinase 3, also known as *ERK1*) and *TGFB1* (transforming growth factor beta 1) signaling (**Figure 4D**), which possibly explains its weaker effects on scratch-wound healing due to attenuated activation of the signal transduction pathways leading to cellular proliferation, differentiation, and growth. As such, direct comparisons between procyanidin B2 and its structural units in equimolar concentrations revealed that epicatechin and its metabolite HPCA were associated with superior scratch-wound healing activity *in vitro*. These results are in agreement with those obtained in previous studies. Epicatechin ester of gallic acid (epicatechin gallate) displayed promising wound healing effects in a 3T3 mouse fibroblast model (Schmidt et al., 2010), as well as *in vivo* in a full-thickness incisional model of wound healing in rats (Kapoor et al., 2004). The predominant green tea polyphenol, epigallocatechin-3-gallate (EGCG), also elicited growth and differentiation of pooled normal human primary epidermal keratinocytes (Hsu et al., 2003). Although the present study did not examine the effects of bioactive structures on epidermal repair functions during wound healing (i.e., keratinocyte proliferation and re-epithelization) based on the above evidence, it is likely that Alaskan berry bioactive compounds could also modulate keratinocyte activities and mediate improvement of epidermal proliferation during re-epithelization, which occurs in conjunction with dermal fibroblast migration and granulation tissue formation to facilitate wound closure. Highlighting their interconnected functionality, fibroblasts and keratinocytes are known to engage in cross-talk, which may act as an important regulator of their respective dermal and epidermal repair functions during skin wound healing.

### Bioenergetic Characterization of Procyanidin B2 and Its Metabolites in Fibroblasts

**Figure 5:**
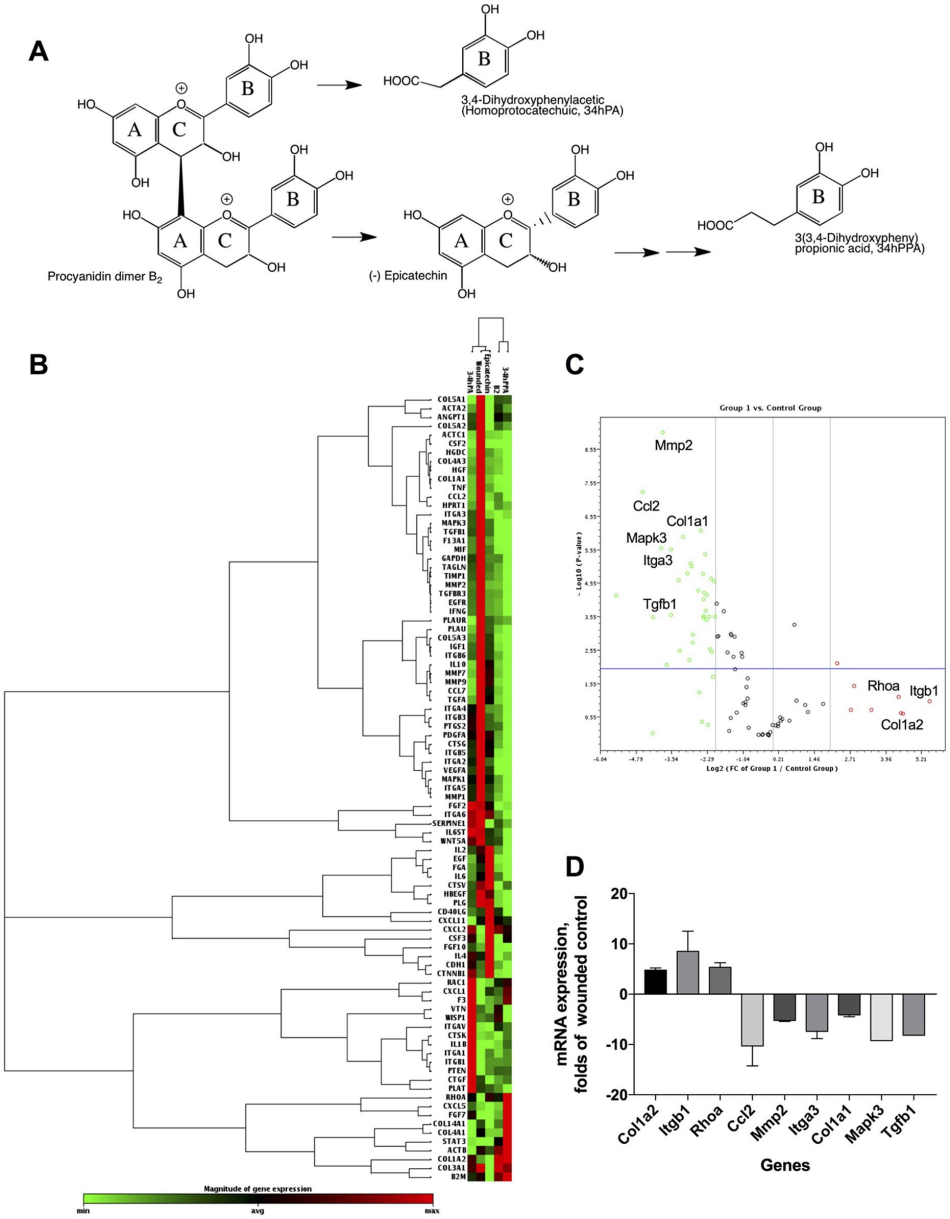
Pharmacogenomic evaluation of scratch-wound healing effects of procyanidin B2, epicatechin, 3,4-dihydroxyphenylacetic acid [(homoprotocatechuic acid (HPCA)], and 3-(3,4-dihydroxyphenyl) propionic acid on gene expression levels in HDFa. **(A)** Schematic pathway of procyanidin B2 hydrolysis into individual structural units. **(B)** Clustergram analysis of quantitative polymerase chain reaction (qPCR) array gene expression profiles in response to scratch-wounding and after 24 h treatment with equimolar concentrations (10 µM) of procyanidin B2 or its metabolites. **(C)** Volcano plot between the combined qPCR array gene expression profile of procyanidin B2 and metabolites and scratch-wounded HDFa cells. **(D)** Changes in mRNA expression levels that responded to all individual compounds tested: *COL1A2*, *ITGB1*, *RHOA*, *MMP2*, and *CCL2*. Only procyanidin B2 showed moderate suppression of *MAPK3* and *TGFB1* expression.

To determine whether the beneficial effects of procyanidin B2 and its structural units on wound healing are mediated by changes in mitochondrial function and bioenergetics, we examined the activity of two major pathways of cellular respiration, glycolysis and oxidative phosphorylation in mitochondria, by directly measuring cellular bioenergetics coupled with mitochondrial stress tests using an XF24 Extracellular Flux Analyzer (**Figure 5**). Under basal conditions, all groups showed comparable OCRs (**Figure 5C**). Treatment with procyanidin B2 elevated fibroblast mitochondrial function without reaching significance. This effect could be largely attributed to its structural subunits, as epicatechin and, to a certain degree, HPCA (3,4-dihydroxyphenylacetic acid) tested in the equimolar concentration produced marked elevation of mitochondrial function by increasing maximal OCR (**Figure 5E**), OCR directed at ATP production (**Figure 5F**), and decreased proton leak (**Figure 5G**). Modification of HPCA to 3-(3,4-dihydroxyphenyl)propionic acid reduced these effects, as chain length extension possibly enabled this compound to reach the mitochondrial membrane and to partially uncouple the mitochondrial electron transport chain. On the other hand, glycolysis was elevated only by procyanidin B2 treatment alone, and this effect could not be attributed to any of its functional groups, suggesting that an intact procyanidin dimer was required for this activity (**Figure 5B**).

Mitochondria have critical roles in regulating cellular metabolism, signaling, ROS production, and calcium homeostasis during tissue repair; productive healing depends on mitochondrial calcium uptake, induced after injury, which consequently stimulates ROS generation, causing RhoA activation and actin accumulation at the wound, which ultimately leads to the initiation of membrane repair (Horn et al., 2017). Our observations in the current work suggest that procyanidin B2 and its bioactive structural metabolites promoted wound repair by stimulating oxidative phosphorylation, thereby increasing mitochondrial ATP synthesis and ROS signaling, which are highly implicated in the initiation of cell repair and regenerative responses at the injury site. We speculate that this, in turn, promoted further association of cellular f-actin filaments with upregulated integrins and induced integrin-dependent fibroblast-like migration (Vicente-Manzanares et al., 2009) as we observed *in vitro*. Mitochondria fundamentally participate in metabolic regulation during tissue repair processes, and it has been suggested that dysfunctional shifts of metabolic programming in immune and nonimmune cells may represent a driving force behind impaired wound healing, failed regenerative outcomes, as well as fibrosis and scarring (Eming et al., 2017). The conserved role of mitochondria in repair and regeneration across many different types of cells and tissues further supports the notion that bioactive polyphenolic compounds of botanical origin, specifically procyanidin B2 and its structural metabolites present in Alaskan wild berries, may promote early phases of wound repair and regeneration, critically dependent on mitochondrial respiration and increased expression of the ECM proteins such as integrins and collagens.

## Discussion

In summary, our results revealed a protective role for polyphenols from three compositionally diverse Alaskan wild berries in signaling and mitochondrial function in cellular wound repair. In general, similar phenolic structures and metabolites are widely distributed across the plant kingdom, which greatly broadens the applicability of the current work. Principal bioactive phenolic components in these berries may possess immunomodulatory properties, encouraging inflammatory resolution and transition across early stages of repair, as well as potentially protecting against proteolytic degradation of ECM, which is often associated with skin aging. Procyanidin B2 and its structural metabolites demonstrated anti-inflammatory activities but appeared to promote wound repair *via* increasing mitochondrial basal respiration, ATP production, and maximum respiratory capacity, potentially contributing to upregulated expression of integrins and other molecules regulating the ECM. Thus, manipulation of mitochondrial function and integrin signaling with epicatechin and HPCA may be of interest in the development of novel wound therapeutics and skincare products aimed at improving skin repair and regenerative functions.

Integrins and their receptors mechanically link ECM and the actin cytoskeleton, therefore playing a fundamental role in maintaining mechanical homeostasis and detecting mechanical properties of the ECM, such as stiffness, in a process known as mechanosensing. Integrin signaling importantly mediates cell–ECM interactions and regulation of ECM remodeling in repair, regeneration, and aging (Humphrey et al., 2014; De Pascalis and Etienne-Manneville, 2017). On the other hand, spatial activation of Rho-family GTPases is involved in immune-competent cell migration to sites of tissue injury *via* chemoattractant cues, highlighting their importance in the infiltration of inflammatory cells such as macrophages during the early inflammatory phase of wound healing (Eming et al., 2017).

The inflammatory response after injury is divided into multiple phases, and ordered progression through these phases is crucial for constructive tissue repair and regeneration of normal tissue architecture (Landen et al., 2016). A preliminary pro-inflammatory phase stimulates an innate immune response to induce infiltration of key inflammatory cells, particularly macrophages, into the wound site. Next, transition to a secondary phase occurs with progressive abatement of pro-inflammatory processes as macrophages switch to reparative phenotypes. In the final phase, termination of the inflammatory response is accomplished through diminishment of macrophages and other inflammatory cells at the wound site through apoptosis and clearance, thus paving the way for transition into the proliferative phase of repair.

Most berry extract and fraction treatments showed moderate effectiveness in inhibiting inflammatory response variables (ROS and NO production) and gene expression (*COX-2* and *iNOS*) in LPS-stimulated macrophages. It is likely that Alaskan berry extracts reduce ROS production in activated macrophages by inducing upregulation of endogenous antioxidant enzyme activities, including glutathione reductase (*GSR*), glutathione s-transferase (*GST*), and superoxide dismutase (*SOD*), which represents a virtually ubiquitous mechanism by which polyphenolic-rich berry extracts are known to provide protection against oxidative stress and redox imbalance (Kelly et al., 2017). Broadly, our observations provide evidence for a reasonable degree of anti-inflammatory bioactivity associated with the majority of tested extract and fraction conditions. However, this was by far the most pronounced in the PAC-enriched fractions. Moreover, the relatively divergent impacts on inflammatory response variables observed with respect to individual extraction/enrichment conditions, and across species, render difficult the discernment of precise structural trends underlying anti-inflammatory potency. Bioactive disparities most likely relate to specific differences in phytochemical profile and quantitative composition of the berries tested in this study. It is generally accepted that the immunomodulatory and/or anti-inflammatory effectiveness associated with any given plant-derived phytochemical mixture is typically determined not only by the presence of specific bioactive constituents and their proportional concentrations but also by the numerous physicochemical interactions that potentially occur between individual bioactive constituents, or between individual constituents and inert components of the plant matrix; while often faithfully presumed to be synergistic or additive in nature, the specific biological consequences of these interactions are not well understood but are appreciated to potentially contribute to bioactivity and target tissue accessibility (Liu, 2004; Phan et al., 2018). 

Alaskan wild berries and other botanicals continue to be held in high regard by Alaska native communities for their unique medicinal benefits as well as nutritive qualities (Flint et al., 2011). Medicinal plant usage encompasses an integral element of the traditional ecological knowledge (TEK) of indigenous Artic communities, and the rich ethnobotanical heritage of indigenous Alaskan communities has largely eluded scientific attention until recently. Moving forward, an overarching scientific priority is the integration of ethnobotanical knowledge with modern biomedical principles, which will likely generate major scientific innovations and lead to novel research avenues in the future (Finn et al., 2017). Interestingly, several Alaskan wild berries tested in this study, lowbush cranberry and bog blueberry, have recently demonstrated benefits *in vitro* for neuronal changes associated with neurodegenerative conditions (Scerbak et al., 2016; Maulik et al., 2018; Scerbak et al., 2018).

Future investigations should explore the influence of 3-D matrices on the effects of these bioactive compounds on fibroblast migration and integrin-mediated mechanosensitive ECM signaling, as these are known to be considerably altered between 2-D and 3-D matrices (Tschumperlin, 2013). Furthermore, it warrants consideration for design for future studies that these experiments were performed in a homogenous cell population, which may not reflect physiological relevance to the same degree as in a hetereogenous co-culture, including keratinocytes, immune cells, and/or endothelial cells, all of which cooperatively coordinate wound healing and tissue repair.

Taken together, these findings point to the attractive possibility of using PAC and bioactives such as epicatechins derived from Alaskan wild berries to promote cellular and molecular responses underpinning regenerative wound healing and constructive tissue repair outcomes. Ultimately, their therapeutic utility in skin repair and regeneration appears to be mediated through several distinct but potentially complementary mechanisms of action, which principally include: immunomodulation to attenuate dysregulated inflammatory response and encourage timely inflammatory resolution; modulation of cell–ECM interaction dynamics *via* integrin signaling to optimize cell behaviors, particularly adhesion and migration, which govern regenerative capacity; and regulation of proteolytic tissue factors that contribute to aberrant ECM remodeling and collagen fragmentation in chronic wounds or aging (Pang et al., 2017).

## Data availability

The raw data supporting the conclusions of this manuscript will be made available by the authors, without undue reservation, to any qualified researcher.

## Ethics Statement

All work was carried out in accordance with Biosafety Level 2 (BSL-2) Biological use authorization (BUA) 2018-11-688.

## Author Contributions

DE and SK contributed the conception and design of the study. DE and SK performed the statistical analysis. DE and JO wrote the first draft of the manuscript. DE, SK, JO, MG, and ML wrote sections of the manuscript. All authors contributed to manuscript revision and read and approved the submitted version.

## Funding

This work is supported by North Carolina State University—Hatch Project no. NC02671 from the United States Department of Agriculture (USDA) National Institute of Food and Agriculture.

## Conflict of Interest Statement

The authors declare that the research was conducted in the absence of any commercial or financial relationships that could be construed as a potential conflict of interest.
