# Supplementary figures and images for: Alaskan Berry Extracts Promote Dermal Wound Repair Through Modulation of Bioenergetics and Integrin Signaling
Source: Front Pharmacol. 2019 Sep 27;10:1058. doi: 10.3389/fphar.2019.01058 (PMC6776586; doi:10.3389/fphar.2019.01058)

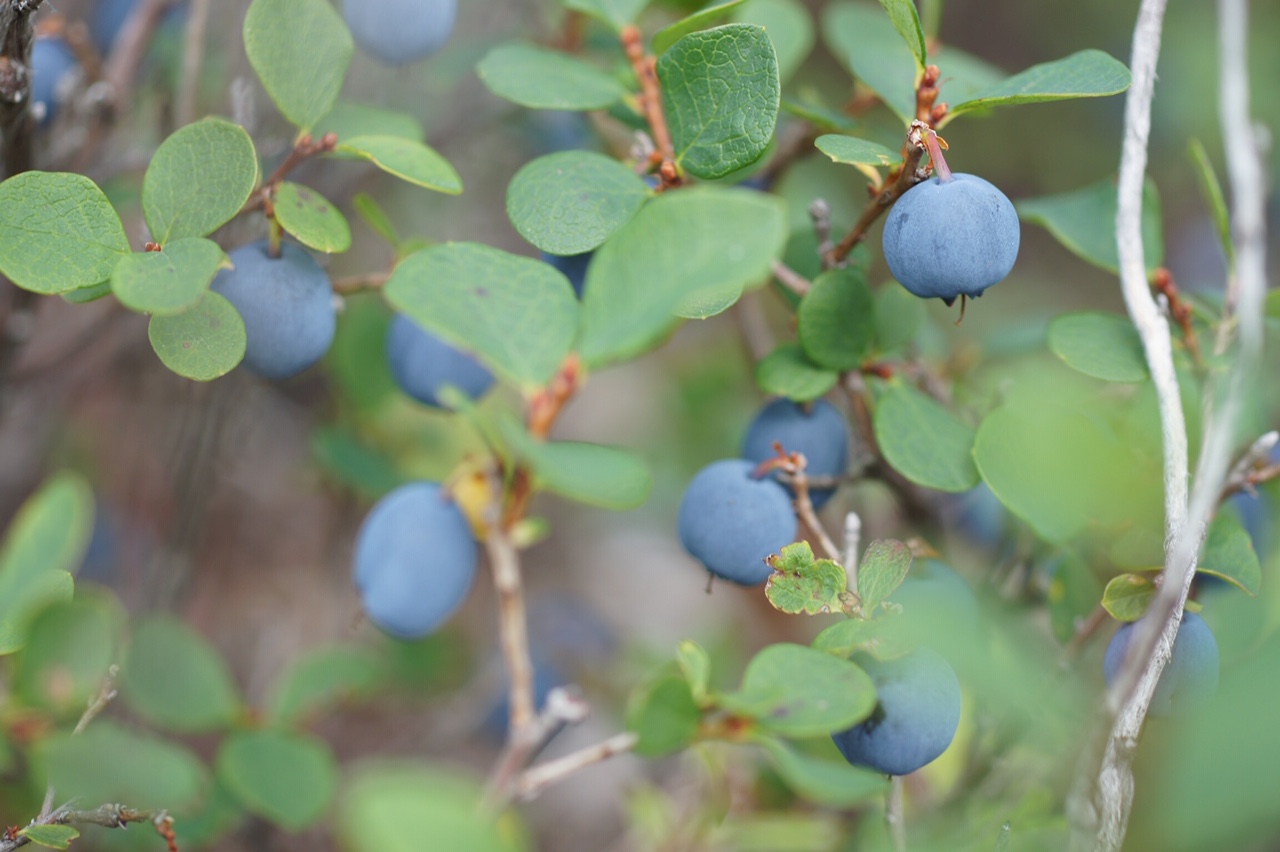

Supplement: Supplementary file 1 [file DataSheet_1.zip › Alaskan Berries Esposito/bog blueberry AK.JPG]

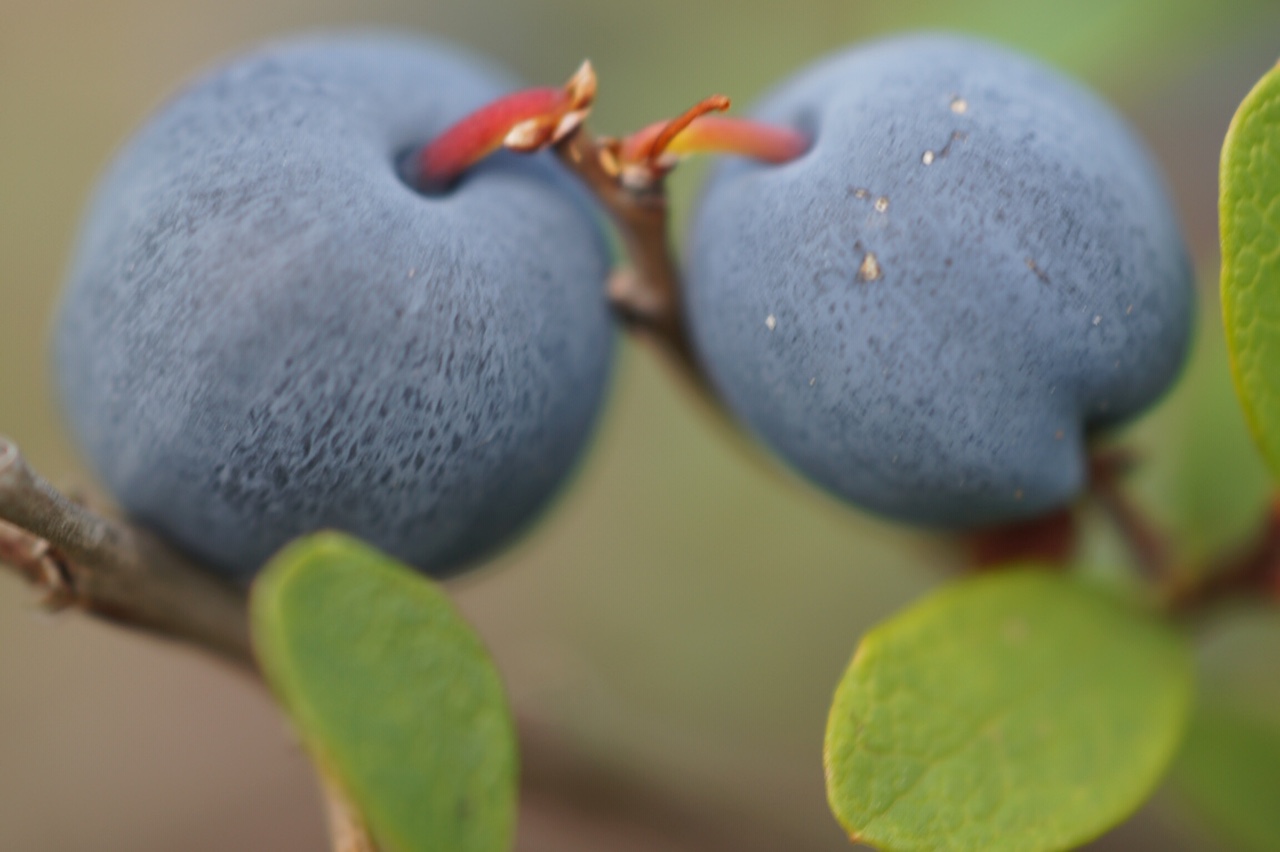

Supplement: Supplementary file 1 [file DataSheet_1.zip › Alaskan Berries Esposito/bog blueberry2 AK.JPG]

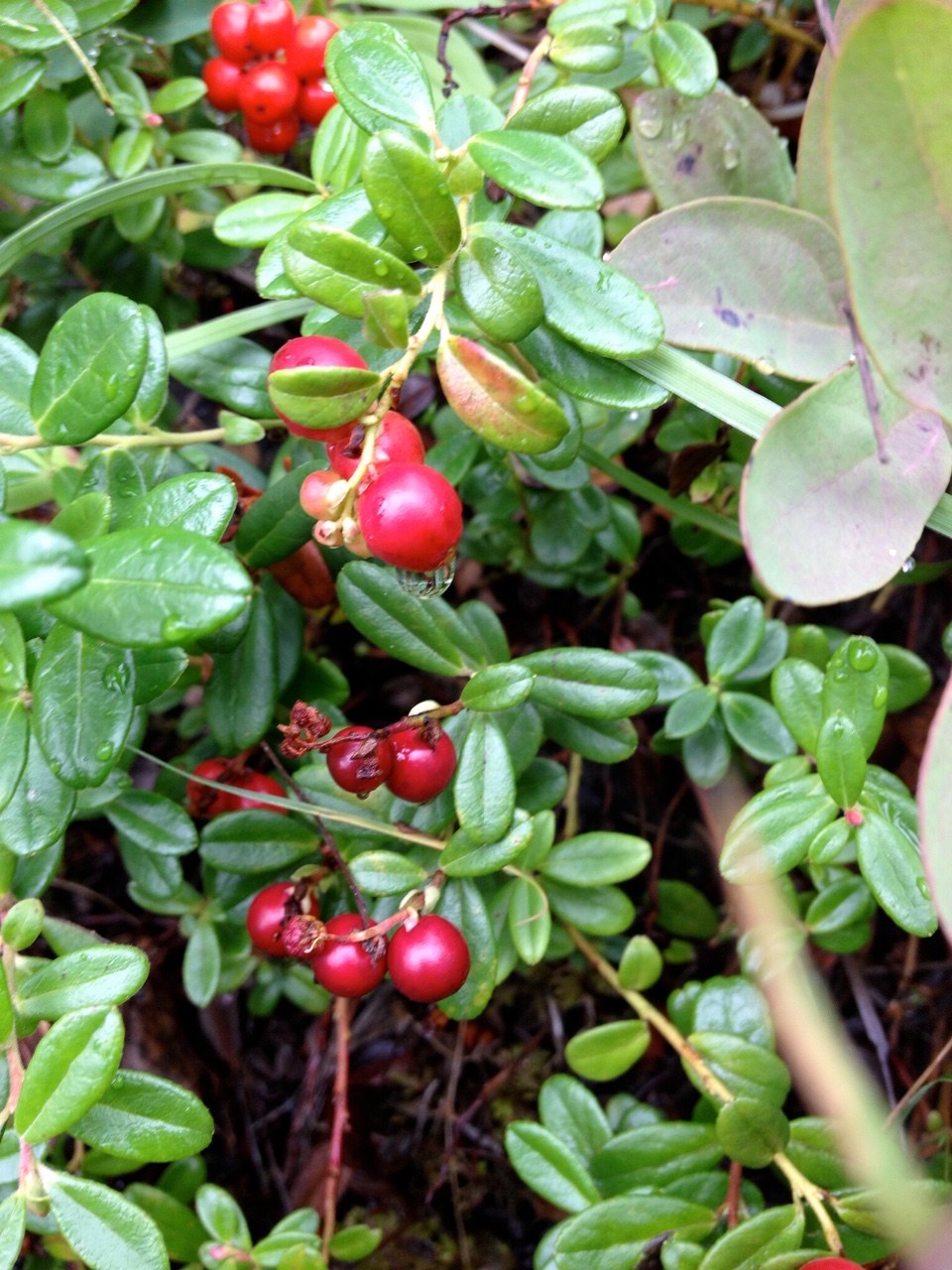

Supplement: Supplementary file 1 [file DataSheet_1.zip › Alaskan Berries Esposito/lowbush cranberry AK.JPG]

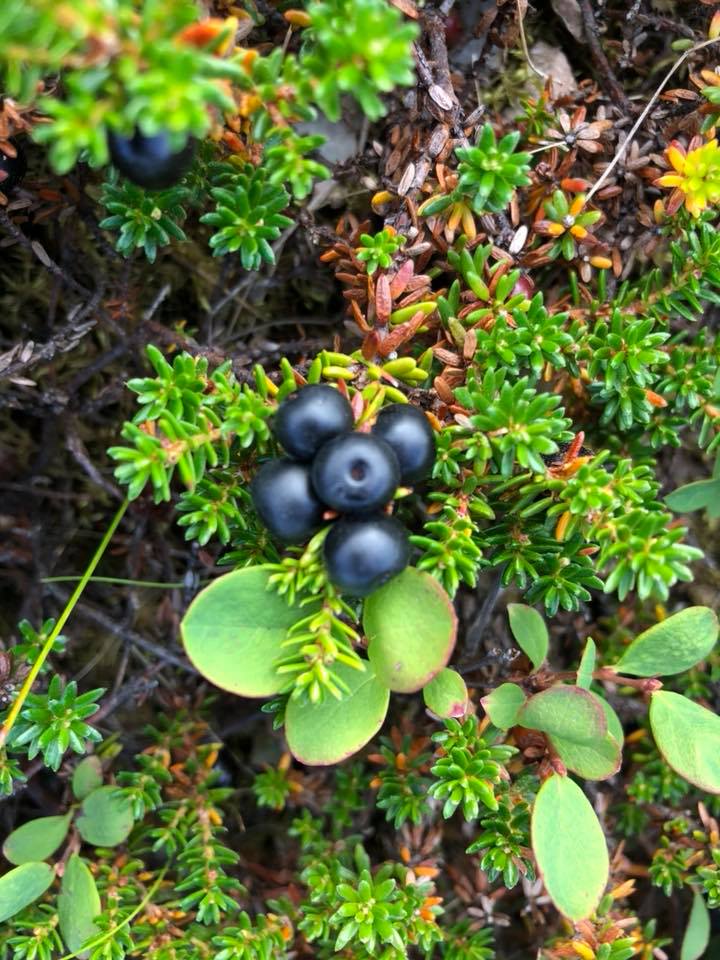

Supplement: Supplementary file 1 [file DataSheet_1.zip › Alaskan Berries Esposito/mossberries crowberries.jpg]

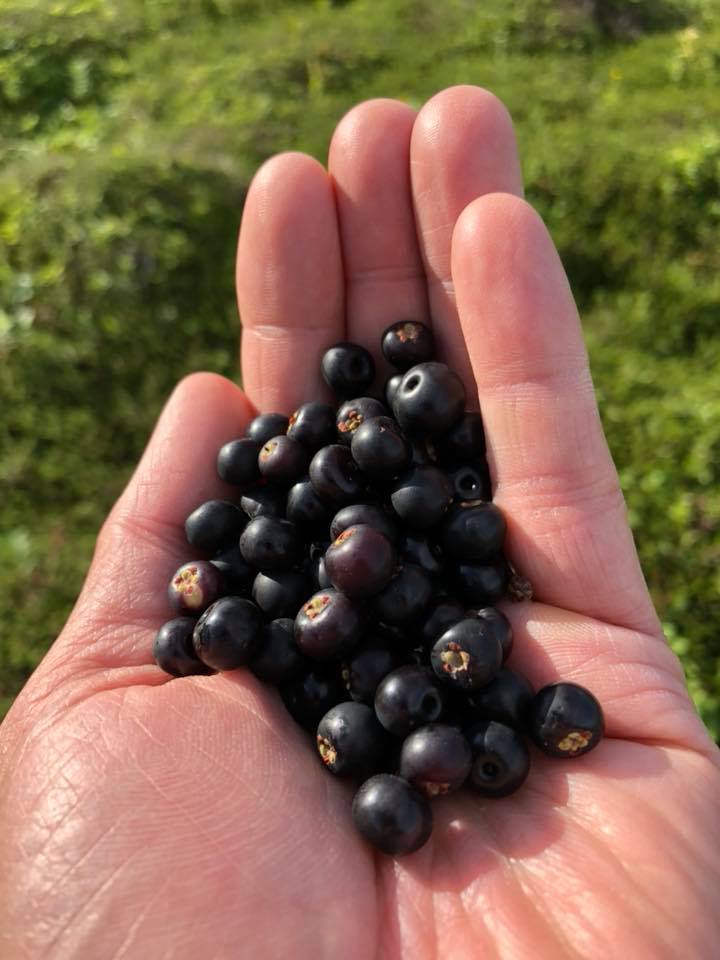

Supplement: Supplementary file 1 [file DataSheet_1.zip › Alaskan Berries Esposito/mossberries in Gary hand.jpg]

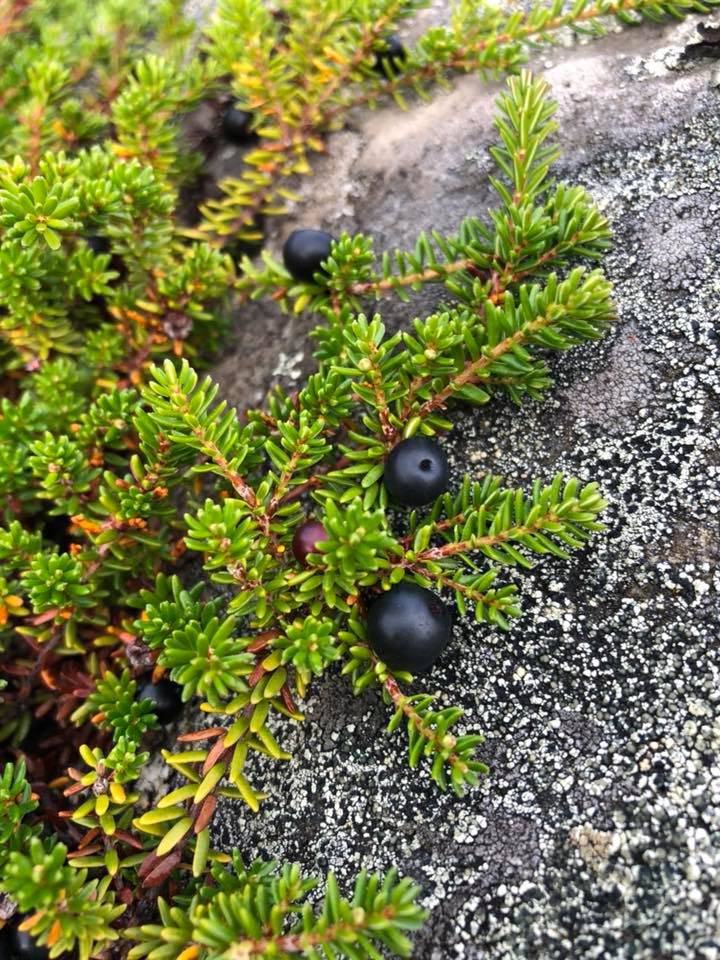

Supplement: Supplementary file 1 [file DataSheet_1.zip › Alaskan Berries Esposito/mossberries.jpg]

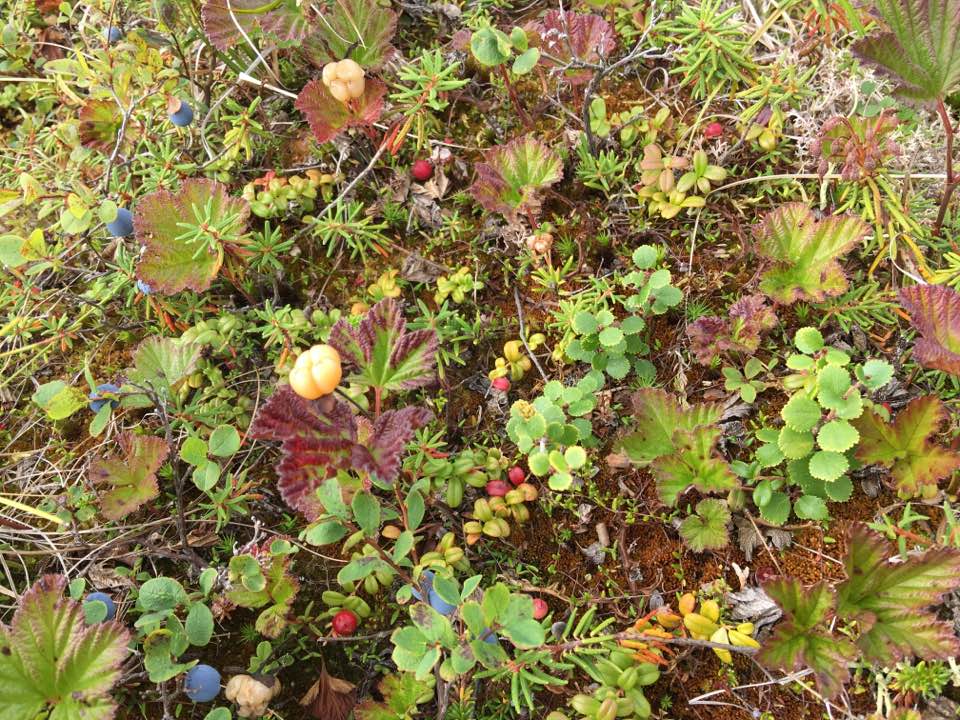

Supplement: Supplementary file 1 [file DataSheet_1.zip › Alaskan Berries Esposito/mixed AK berries (2).jpg]
